# Associations of polyunsaturated fatty acids and genetic predisposition with cardiovascular risk among hypertensive adults

**DOI:** 10.3389/fnut.2025.1623962

**Published:** 2025-10-22

**Authors:** Jindi Li, Yubo Zhang, Shaohui Liu, Cuihua Nong, Quanhong Chen, Yunfeng Zou, Yang Peng, Hao Chen

**Affiliations:** ^1^Department of Toxicology, School of Public Health, Guangxi Medical University, Nanning, China; ^2^Department of Occupational and Environmental Health, School of Public Health, Guangxi Medical University, Nanning, China

**Keywords:** omega-3 polyunsaturated fatty acids, omega-6 polyunsaturated fatty acids, hypertension, polygenic risk scores, cardiovascular disease

## Abstract

**Background:**

Hypertension is linked to elevated cardiovascular morbidity and mortality. Research findings regarding cardiovascular benefits of polyunsaturated fatty acids (PUFAs) are inconsistent, possibly due to unbalanced N6FA/N3FA (omega-6 to omega-3 polyunsaturated fatty) ratios and genetic predispositions in PUFAs utilization and cardiovascular disease (CVD) risk. This study investigates the association between plasma PUFAs and CVD risk among hypertensive adults, stratified by the polygenic risk score (PRS) for PUFAs and CVD.

**Methods:**

The study analyzed 135,969 hypertensive adults from the UK Biobank. Cox regression models were employed to assess the links between PUFAs and cardiovascular outcomes, as well as the moderating effect of PRS.

**Results:**

During the follow-up, 22,084 (16.2%) of participants experienced CVD events, and 2,336 (1.7%) and 13,823 (10.2%) died from CVD and all causes, respectively. Higher blood levels of total polyunsaturated fatty acids (Total PUFA), N3FA, N6FA, docosahexaenoic acid (DHA), and linoleic acid (LA) were associated with lower CVD incidence and mortality, for instance, the hazard ratio for N3FA was 0.745 [95% confidence interval (0.698, 0.796)]. Furthermore, the N6FA/N3FA ratio showed a positive association with CVD incidence and mortality, with the point of minimum risk estimated at approximately 8.70 based on restricted cubic spline analysis. Protective associations of Total PUFA, N6FA, and LA with CVD incidence were stronger in individuals with lower CVD – PRS scores.

**Conclusion:**

Despite the general cardiovascular benefits of PUFAs, a higher N6FA/N3FA ratio was associated with an elevated risk of CVD in hypertensive participants. The benefits of PUFAs are greater in those with lower genetic CVD risk. This emphasizes the need to consider N6FA/N3FA balance and genetic predisposition when assessing health impact of PUFAs on CVD.

## Background

Over 1 billion people globally have hypertension (1), with its prevalence and related disease burden continuously increasing. Hypertension accounts for approximately 10% of the global healthcare expenditure, posing significant health and economic burden (2). Hypertension is a major risk factor for elevated morbidity and mortality of cardiovascular diseases (CVD), such as coronary artery disease, heart failure, and stroke (3). Studies have shown that long-term hypertension aggravates vascular hardening (4), promotes oxidative stress and inflammatory responses (5), and leads to changes in cardiac structure by increasing the pressure load on the heart and blood vessels. Previous studies indicated that higher intake of omega-3 fatty acids may result in reductions in all-cause mortality and potential cardiovascular protection among patients with diabetes, it is possible to hypothesize that the protective effects are likely to be present in patients with hypertension (6). Lowering CVD risk among hypertensive adults bears great clinical and public health significance.

Polyunsaturated fatty acids (PUFAs), including omega-3 polyunsaturated fatty acids (N3FA) and omega-6 polyunsaturated fatty acids (N6FA), are promoted for their cardiovascular benefits (7, 8). For instance, N3FA including eicosapentaenoic acid (EPA) and docosahexaenoic acid (DHA), mainly from marine fish, lowers CVD risk through mechanisms such as enhancing vascular function, modulating the autonomic nervous system, reducing arterial inflammation, and stabilizing heart rhythms (9, 10). Although dietary intake of N3FA is believed to have cardiovascular benefits, recent randomized clinical trials and large-scale meta-analyses have failed to substantiate the protective effects of N3FA supplementation on CVD (11, 12). The possible causes of these inconsistencies may stem from two reasons, the imbalanced N6FA/N3FA ratio and individual genetic susceptibility.

Moderate consumption of N6FA, such as linoleic acid (LA) found in vegetable oil, supports brain health and skin integrity but excessive intake may increase thrombosis and inflammation risks detrimental to cardiovascular health (13, 14). Studies show that a lower N6FA/N3FA ratio correlates with reduced chronic disease risk and mortality rates from CVD and cancer (15, 16). A balanced intake of N6FA to N3FA (N6FA/N3FA) ratio is vital for optimal cardiovascular health. A study discovered that in the typical Western diet nowadays, the ratio of N6FA to N3FA is approximately 20:1, with N6FA being predominant. This would render people in a state prone to pro-inflammatory, pro-allergic, pro-thrombotic and autoimmune-susceptible conditions (17). We have previously shown that dietary intake of N3FA is associated with lower CVD morbidity and mortality among US hypertensive adults (18). However, whether the optimal N6FA/N3FA ratio bears similar CVD benefits among hypertensive adults remains undisclosed.

Additionally, the health benefits of PUFAs against CVD may be dependent on the individual genetic predisposition of PUFAs utilization or CVD susceptibility (19). PUFAs will need biochemical processes to be absorbed, metabolized, and utilized by the organisms (20). However, such a process may be dominated by genetic characteristics. For instance, lower metabolic rate of PUFAs due to genetic makeup may result in insignificant health benefit (21). Furthermore, genetic susceptibility of CVD among individuals might also give rise to disparities in the protective effect of PUFAs on CVD (22). It is possible that the genetic predisposition mentioned above may moderate the protective effects of PUFAs on CVD morbidity and mortality among hypertensive adults. The polygenic risk score (PRS) is a genomic assessment tool that calculates an individual’s disease risk based on genetic variants identified in genome-wide association studies (GWAS) (23). We employed this scoring system to assess genetic risk related to PUFAs as well as CVD in this study.

In the current study, by utilizing a prospective cohort from the UK Biobank, we aimed to investigate the associations between plasma PUFAs levels as well as N6FA/N3FA and CVD morbidity and mortality among hypertensive adults. We also examined the moderating effects of PUFA – PRS and CVD – PRS on the associations mentioned above.

## Methods

### Study design and population

The UK Biobank enlisted more than 500,000 participants aged 40 and above from 22 centers in England, Scotland, and Wales during the period from 2006 to 2010. Data on biometrics, lifestyle choices, health status, dietary habits, physical activity levels, and medication usage were collected through face-to-face interviews and self-administered questionnaires. The study was approved by the Northwest Multi-Center Research Ethics Committee, with all participants providing informed consent by signing a form. The current prospective cohort study was conducted under the UK Biobank resource, application number [Project ID: 97753].

**Figure 1:**
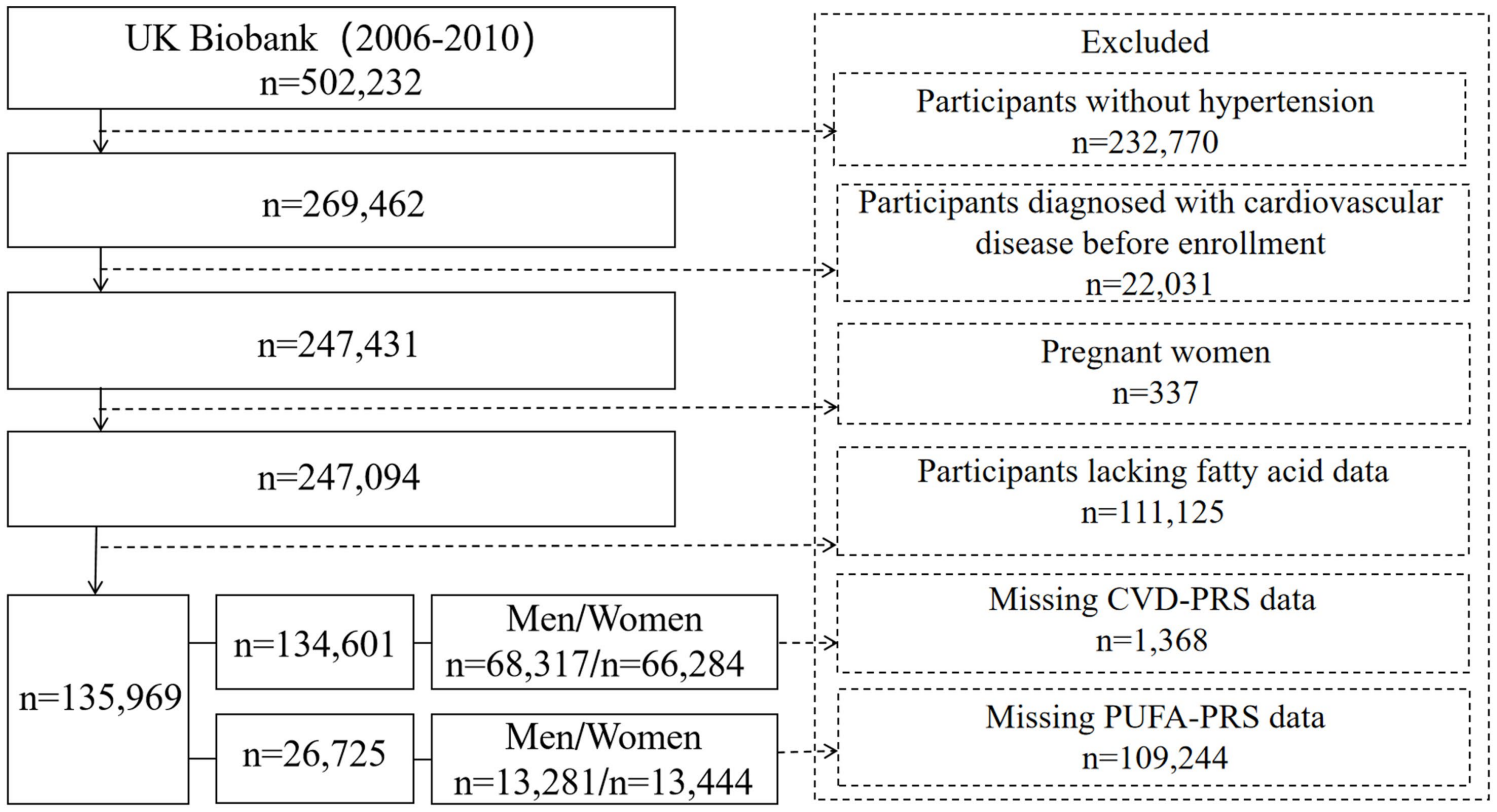
Flow chart of sample selection.

In this study, we included participants with hypertension at the baseline (*n* = 269,462) (Figure 1). Hypertension was defined by meeting one of the following criteria: systolic blood pressure ≥ 140 mmHg or diastolic blood pressure ≥ 90 mmHg at the baseline; use of antihypertensive medications; reporting having hypertension by participants; or a formal diagnosis by a healthcare professional. Hypertensive participants were further excluded based on the following criteria: (1) with at least one CVD at the baseline including myocardial infarction, heart failure, atrial fibrillation, stroke, atherosclerosis, aneurysm, arteriovenous embolism or thrombosis, hypertensive heart disease (*n* = 22,031); (2) pregnant women (*n* = 337); and (3) lacking data on PUFAs (*n* = 111,125). This exclusion resulted in approximately 135,969 participants included in the analysis on the association between PUFAs and CVD morbidity and mortality. To further assess the moderating role of genetic susceptibility on the associations mentioned above, we further excluded participants missing CVD – PRS information (*n* = 1,368) and PUFA – PRS data (*n* = 109,244).

### Assessment of plasma fatty acid levels

In the UK Biobank, the fatty acids levels were detected during the initial assessment phase (2006–2010, i.e., the baseline phase) and the first repeat assessment (2012–2013). In this study, the main analysis was conducted using the plasma fatty acids levels detected in the baseline, while the subsequent sensitivity analysis—time-dependent model—was performed using the fatty acid data from both the baseline and the first repeat measurement. From June 2019 to April 2020 (first phase) and from April 2020 to June 2022 (second phase), a total of 251 metabolic biomarkers—including lipoproteins, fatty acids, the composition of fatty acids, and various low molecular weight metabolites—were measured among approximately 280,000 participants using a high-throughput nuclear magnetic resonance (NMR) analysis platform developed by Nightingale Health Ltd. In our analysis, we included data of the plasma concentrations of Total PUFA, N3FA, N6FA, the ratio of N6FA to N3FA, DHA and LA. Dataset regarding plasma levels of EPA was not available in the UK Biobank.

### Assessment of health outcomes

All participants consented to long-term follow-up through electronic health records, which include hospital records (managed by Hospital Episode Statistics and Scottish Morbidity Records under the Ministry of Health), death certificates, and cancer registries (administered by the Office for National Statistics and General Register Office). Each record was classified according to the International Statistical Classification of Diseases and Related Health Problems 10th Revision (ICD-10). The present study focused on the following health outcomes:

- CVD events. CVD events included myocardial infarction, heart failure, atrial fibrillation and flutter, stroke, atherosclerosis, aneurysm, arteriovenous thrombosis or embolism, hypertensive heart disease. The relevant ICD-10 codes are I11, I21, I22, I48, I50, I64, I70-I72, and I74-I82.
- All-cause and CVD mortality. Data of all-cause deaths and cardiovascular deaths were identified and individuals who survived were censored on December 19, 2022. Follow-up time was quantified in person-years from the date of participant enrollment until either CVD diagnosis or death, depending on which event appeared first. The corresponding ICD codes are: I05, I07-I08, I10-I11, I13, I20-I21, I24-I25, I30-I31, I33-I35, I38, I42, I45-I51, I64, I69-I74, I77-I78, I80, I82-I83, I85, I87.

### Assessment of polygenic risk score

To assess genetic susceptibility, we utilized the PRSs developed from the UK Biobank, which aggregate genome-wide data on the associations between single-nucleotide polymorphisms (SNPs) and specific phenotypes. These PRSs provide a composite measure of an individual’s genetic predisposition to certain diseases or traits. The PRSs from the UK Biobank are based on genome-wide association study (GWAS) data and are tailored for participants of diverse ancestries. The UK Biobank has released optimized PRS for 53 diseases and quantitative traits, with systematic evaluations of related phenotypes and genetic information available in the src/ukb_pret/data/traits.yaml file. We obtained these CVD – PRS from the UKB data-field 26,224 and obtain PUFA – PRS from UKB data field 26,264, 26,256, 26,257 and 26,231. For our analysis, PRS scores for CVD, Total PUFA, N3FA, N6FA, and DHA were dichotomized based on the median to categorize individuals as having either “low” or “high” genetic risk.

### Covariates

Covariates included age, sex, ethnicity, smoking status, alcohol consumption, education level, household income, Townsend Deprivation Index, physical activity [data on metabolic equivalent (MET) scores derived from the International Physical Activity (IPAQ) guidelines], body mass index (BMI), antihypertensive medication use, total serum cholesterol levels, chronic renal dysfunction or decreased eGFR, and diabetes. The covariate information was collected at the baseline through touchscreen questionnaires, verbal interviews, or various tests. Ethnicity was classified as white or other. Smoking state included never smoker, former smoker, and current smoker. Drinking state was categorized into never drinker, former drinker, and current drinker. Education levels were classified into three levels: low, middle, and high. High level of education means having a College or University degree, or other professional qualifications such as nursing or teaching. Middle education level refers to A levels/AS levels, O levels/GCSEs, CSEs, or equivalent qualification. A low educational level refers to an NVQ, HND, HNC, or equivalent qualification. Physical activity levels were classified as high, moderate or low. Household income before tax was divided into less than £31,000; between £31,000 to £51,999; or greater than £52,000. The Townsend Deprivation Index—based on national census data—measures socioeconomic status using factors including car ownership, household overcrowding, head of household occupation, and unemployment rates—with higher scores indicating greater social deprivation. Body mass index (BMI) was calculated using the formula [weight (kg)/height (m)^2^]. Antihypertensive medication usage was recorded as binary: yes or no. Chronic renal dysfunction is defined as the first reported diagnosis, decreased eGFR value defined as less than 60 mL/min/1.73 m^2^, calculated using the Modification of Diet in Renal Disease (MDRD) formula (24). The diagnosis of diabetes was based on the participants’ self-reported disease information, and the usage was recorded in a binary form: yes or no. Total serum cholesterol levels were analyzed as a continuous variable.

### Statistical analysis

Continuous variables were presented as mean ± standard deviation (SD), while categorical variables were expressed as count (percentage). Plasma concentrations of PUFAs were reported in mmol/L. We employed PUFAs concentrations as both continuous and categorical data in the statistical models. Using a Cox proportional hazards regression model, we estimated associations of PUFAs with CVD morbidity and mortality, reporting hazard ratios (HRs) with 95% confidence intervals (CIs) for each exposure quartile. All the models were adjusted for age, sex, race, smoking and drinking status, education level, household income, Townsend Deprivation Index, physical activity, body mass index (BMI), antihypertensive medication use, total serum cholesterol levels, chronic renal dysfunction or decreased eGFR, and diabetes.

To explore the impact of genetic susceptibility on the links between PUFAs and CVD morbidity and mortality, we performed regression analyses with multiplicative terms between PUFAs and their corresponding PUFA – PRS or between PUFAs and CVD – PRS. Additionally, we assessed whether the associations between PUFAs and CVD morbidity and mortality varied by PRS category (high vs. low). We conducted a subgroup analysis by sex to examine the associations between PUFAs and CVD morbidity and mortality. We adopted a time-dependent Cox model and verified the robustness of these results by measuring PUFAs at two different time points. To address reverse causality, we excluded those who had experienced CVD events before baseline (2006–2010) and those who had CVD events between baseline and the first repeat (2012–2013). All analyses were performed using R Studio version 4.4.1, with a two-sided *p* < 0.05 set statistically significant.

## Results

### Participant characteristics at the baseline

The analysis comprised 135,969 hypertensive participants, with a mean age of 58.6 years (SD: 7.42). More than half of the participants were male (50.7%), white (95.1%), identified as having at least a secondary education (71%), reporting a household income below £31,000 (52.3%), engaging in moderate to vigorous physical activity (80.8%), and current alcohol consumers (92.2%), current smokers (53.5%). Diagnosed with diabetes (7.2%), and suffering from Chronic renal dysfunction or decreased eGFR (18.2%), and taking antihypertensive medications (35.4%). The mean values for BMI, total serum cholesterol level, and Townsend Deprivation Index were 28.5 (4.9) kg/m^2^, 5.7 (1.6) mg/dL, and −2.2 (4.0), respectively (Table 1). In addition, Total PUFA was divided into high and low to describe the baseline characteristics of the participants.

**Table 1:** Characteristics of hypertensive participants in UK Biobank.

Variables	Total (*n* = 135,969)	Total PUFA	
		High (*n* = 67,985)	Low (*n* = 67,984)
Age, years, Mean (SD)	58.6 (7.42)	58.8 (7.10)	58.3 (7.71)
Sex, *n* (%)			
Men	69,000 (50.7)	27,840 (40.9)	41,160 (60.5)
Women	66,969 (49.3)	40,145 (59.1)	26,824 (39.5)
Race/ethnicity, *n* (%)			
White	129,371 (95.1)	64,951 (95.5)	64,420 (94.8)
Other	6,598 (4.9)	3,034 (4.5)	3,564 (5.2)
Education level, *n* (%)			
High	45,939 (33.7)	23,092 (33.9)	22,847 (33.6)
Moderate	50,837 (37.3)	25,961 (38.1)	24,876 (36.5)
Low	10,332 (7.5)	4,689 (6.8)	5,643 (8.3)
Annual household income, *n* (%)			
<£31,000	71,177 (52.3)	35,727 (52.6)	35,450 (52.1)
£31,000 ~ £51,999	22,604 (16.6)	11,182 (16.4)	11,422 (16.8)
≥£52,000	42,188 (31.1)	21,076 (31.0)	21,112 (31.1)
Smoking status, *n* (%)			
Previous	50,353 (37.0)	25,015 (36.8)	25,338 (37.3)
Current	72,781 (53.5)	37,200 (54.7)	35,581 (52.3)
Never	12,835 (9.4)	5,770 (8.5)	7,065 (10.4)
Alcohol status, *n* (%)			
Previous	5,953 (4.3)	2,902 (4.2)	3,051 (4.5)
Current	125,422 (92.2)	63,121 (92.9)	62,301 (91.6)
Never	4,594 (3.3)	1,962 (2.9)	2,632 (3.9)
IPAQ activity group, *n* (%)			
High	55,384 (40.7)	28,155 (41.4)	27,229 (40.1)
Moderate	54,457 (40.1)	27,465 (40.4)	26,992 (39.7)
Low	26,128 (19.2)	12,365 (18.2)	13,763 (20.2)
BMI, kg/m^2^, Mean (SD)	28.5 (4.9)	28.2 (4.7)	28.8 (5.2)
Diabetes, *n* (%)			
Yes	9,855 (7.2)	2,385 (3.5)	7,470 (11.0)
No	126,114 (92.8)	65,600 (96.5)	60,514 (89.0)
Taking anti-hypertensive medication, *n* (%)			
Yes	48,132 (35.4)	19,856 (29.2)	28,276 (41.6)
No	87,837 (64.6)	48,129 (70.8)	39,708 (58.4)
Chronic renal dysfunction or decreased eGFR, *n* (%)			
Yes	24,795 (18.2)	10,366 (15.2)	14,429 (21.2)
No	111,174 (81.8)	57,619 (84.8)	53,555 (78.8)
Total serum cholesterol level, mg/dL, Mean (SD)	5.7 (1.6)	6.4 (1.0)	5.1 (0.9)
Townsend deprivation index, Mean (SD)	−2.2 (4.0)	−1.5 (3.0)	−1.3 (3.1)

Categorical variables were expressed as number (*n*) and weighted percent (%) of the participants. Continuous variables were expressed as weighted median and SD (Standard Deviation). BMI, body mass index. IPAQ, the International Physical Activity Questionnaire.

The median follow-up duration for all participants was 13.8 years. As of December 19, 2022, 22,084 hypertensive patients (16.2%) included in the study experienced CVD events; 2,336 patients (1.7%) died to CVD as the primary cause of death; and 13,823 individuals (10.2%) had died from all causes. Furthermore, the baseline characteristics of hypertensive participants with complete CVD – PRS (*n* = 134,601) and PUFA – PRS (i.e., Total PUFA, N3FA, N6FA, DHA) (*n* = 26,725) were presented in Supplementary Tables S1, S2, respectively. The baseline characteristics of the sub-groups of participants were similar to the ones described above.

### Blood fatty acid levels

The quantitative data for N3FA and N6FA blood levels were aggregated to derive the Total PUFA values. In hypertensive participants (*n* = 135,969), the mean (SD) blood levels of Total PUFA, N3FA, N6FA, DHA, and LA were 5.1 (0.82), 0.55 (0.23), 4.55 (0.7), 0.24 (0.09), and 3.48 (0.7), all units in mmol/L, respectively (Table 2). The mean (SD) for N6FA/N3FA ratio was 9.56 (4.32). In addition, PUFAs were divided into high and low and then calculated the mean and SD. Among participants with PRS scores for PUFA and CVD, the average levels of the fatty acid parameters were similar to all hypertensive participants (Supplementary Tables S3, S4).

**Table 2:** Descriptive statistics of blood fatty acid levels in hypertensive adults.

Variables	Total (*n* = 135,969)	Total PUFA	
		High (*n* = 67,985)	Low (*n* = 67,984)
Total PUFA, mmol/L	5.1 (0.82)	5.75 (0.57)	4.46 (0.43)
N3FA, mmol/L	0.55 (0.23)	0.72 (0.19)	0.38 (0.09)
N6FA, mmol/L	4.55 (0.7)	5.10 (0.49)	4.00 (0.37)
N6FA/N3FA, ratio	9.56 (4.32)	12.32 (4.52)	6.81 (1.31)
DHA, mmol/L	0.24 (0.09)	0.30 (0.07)	0.17 (0.04)
LA, mmol/L	3.48 (0.7)	4.03 (0.50)	2.93 (0.37)

Data were presented as mean (Standard Deviation). DHA, docosahexaenoic acid; LA, Linoleic acid; N3FA, n-3 polyunsaturated fatty acid; N6FA, n-6 polyunsaturated fatty acid; Total PUFA, N6FA + N3FA.

### Associations between PUFAs and CVD morbidity, CVD mortality, and all-cause mortality

**Figure 2:**
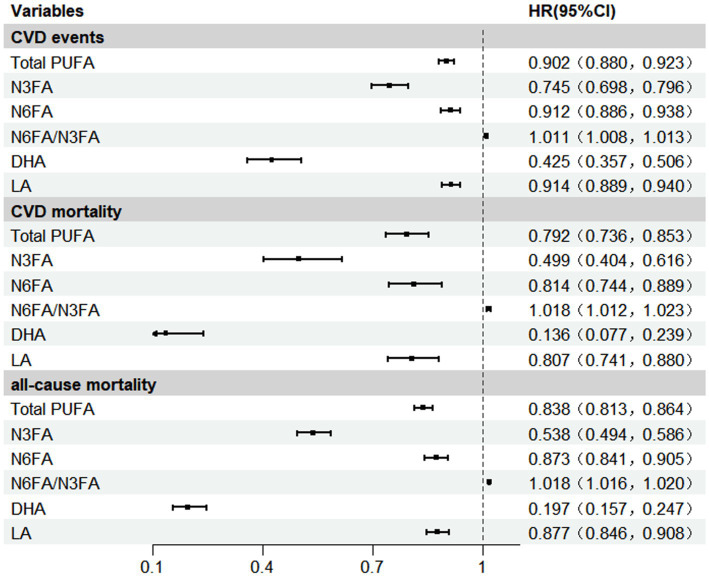
Associations between PUFAs and CVD events, CVD mortality, and all-cause mortality. Model was adjusted for covariates including age, gender, race, educational level, income, body mass index, smoking, alcohol, IPAQ activity group, diabetes, townsend deprivation index, total serum cholesterol level, taking anti-hypertensive medication, chronic renal dysfunction or decreased eGFR. DHA, docosahexaenoic acid; IPAQ, the International Physical Activity Questionnaire; LA, Linoleic acid; N3FA, n-3 polyunsaturated fatty acid; N6FA, n-6 polyunsaturated fatty acid; Total PUFA, N6FA + N3FA.

After adjustment for potential confounding factors, Total PUFA were associated with lower risk of CVD events [HR (95% CI): 0.902 (0.880, 0.923)], CVD-related mortality [0.792 (0.736, 0.853)], and all-cause mortality [0.838 (0.813, 0.864)], respectively. We observed that higher blood N3FA concentrations were associated with lower risk of CVD events [HR (95% CI): 0.745 (0.698, 0.796)], CVD-related mortality [0.499 (0.404, 0.616)], and all-cause mortality [0.538 (0.494, 0.586)], respectively. Similarly, we observed lower HRs for CVD incidence, CVD mortality, and all-cause mortality associated with blood levels of N6FA [0.912 (0.886, 0.938), 0.814 (0.744, 0.889), and 0.873 (0.841, 0.905)], DHA [0.425 (0.357, 0.506), 0.136 (0.077, 0.239), and 0.197 (0.157, 0.247)], and LA [0.914 (0.889, 0.940), 0.807 (0.741, 0.880), 0.877 (0.846, 0.908)], respectively (Figure 2).

Compared to the lowest quartile (Q1), participants in Q4 levels of total PUFA, N3FA, N6FA, DHA, and LA were associated with lower risk of CVD events, CVD mortality, and all-cause mortality. For example, compared with Q1, participants in Q4 showed lower risk of CVD incidence [0.839 (0.797, 0.883)], CVD mortality [0.665 (0.567, 0.780)], and all-cause mortality [0.730 (0.684, 0.779)], respectively (Supplementary Table S5).

In contrast, increased plasma N6FA/N3FA exhibited a slightly elevated risk for CVD events [HR (95% CI): 1.011 (1.008, 1.013), CVD-related mortality 1.018 (1.012, 1.023), and all-cause mortality 1.018 (1.016, 1.020)], respectively (Figure 2). Based on restricted cubic spline analysis, our study suggests that the risk of CVD events, CVD-related mortality, and all-cause mortality begins to rise markedly as the N6FA/N3FA ratio increases beyond approximately 8.70, which corresponded to the point of lowest risk in our cohort, *P* for non-linearity <0.001 (Supplementary Figures S1–S3). Compared to the lowest quartile (Q1), blood levels of N6FA/N3FA were associated with elevated risk of CVD events [HR _Q4 vs. Q1_ (95% CI): 1.156 (1.113, 1.201), CVD-related mortality 1.364 (1.214, 1.533), all-cause mortality 1.366 (1.303, 1.433)], respectively. Apart from the results, the reduction or increase in risks of CVD events, CVD-related deaths, and all-cause deaths associated with Total PUFA, N3FA, N6FA, N6FA/N3FA, DHA and LA would be intensified along with the increase in the concentrations of the corresponding plasma PUFAs (*P* for trend <0.001, respectively) (Supplementary Table S5).

### Moderating effects of PRS on the associations between PUFAs and CVD health outcomes

We investigated the interactive effects of PUFAs and PRS associated with either PUFA or CVD susceptibility among hypertensive individuals in the UK Biobank. Compared with participants with low CVD – PRS, the HR of CVD incidence related to plasma Total PUFA concentration were slightly higher among those with high CVD – PRS [low vs. high, HR (95% CI): 0.879 (0.848, 0.912) vs. 0.921 (0.891, 0.951), *P*_interaction_ = 0.0097]. Similarly, compared with the high CVD-PRS group, we observed more prominent protective associations of N6FA [low vs. high: 0.894 (0.856, 0.934) vs. 0.927 (0.892, 0.964), *P*_interaction_ = 0.016] and LA [low vs. high, HR (95% CI): 0.899 (0.862, 0.937) vs. 0.927 (0.893, 0.963), *P*_interaction_ = 0.007] with CVD incidence in the low group (Table 3). On the contrary, N6FA/N3FA was associated with a higher risk of CVD incidence [low vs. high: 1.015 (1.011, 1.020) vs. 1.008 (1.005, 1.011), *P*_interaction_ = 0.026] and all-cause mortality [low vs. high: 1.025 (1.020, 1.030) vs. 1.016 (1.014, 1.019, *P*_interaction_ = 0.0017)] in individuals with a low CVD – PRS compared with those with a high score (Table 3). We also examined the effects of PUFAs, PUFA – PRS, and their interactions on CVD events, CVD-related deaths, and all-cause deaths. There was no statistically significant interaction between plasma Total PUFA, N3FA, N6FA, DHA, and PUFA – PRS on CVD events, CVD-related deaths, and all-cause deaths (Supplementary Table S6).

**Table 3:** Interactions between blood levels of PUFAs and CVD-related PRS on CVD events, CVD mortality, all-cause mortality in hypertensive adults.

Variables	Hazards ratio (95%CI)		*P_interaction_*
	Low (CVD – PRS)	High (CVD – PRS)	
CVD events			
Total PUFA	0.879 (0.848, 0.912)***	0.921 (0.891, 0.951)***	0.010
N3FA	0.686 (0.621, 0.757)***	0.798 (0.731, 0.872)***	0.053
N6FA	0.894 (0.856, 0.934)***	0.927 (0.892, 0.964)***	0.016
N6FA/N3FA	1.015 (1.011, 1.020)***	1.008 (1.005, 1.011)***	0.026
DHA	0.375 (0.289, 0.487)***	0.472 (0.373, 0.597)***	0.354
LA	0.899 (0.862, 0.937)***	0.927 (0.893, 0.963)***	0.007
CVD mortality			
Total PUFA	0.790 (0.704, 0.888)***	0.789 (0.716, 0.869)***	0.660
N3FA	0.558 (0.403, 0.771)***	0.455 (0.343, 0.602)***	0.465
N6FA	0.796 (0.692, 0.915)**	0.821 (0.731, 0.922)***	0.474
N6FA/N3FA	1.019 (1.006, 1.032)**	1.017 (1.011, 1.022)***	0.830
DHA	0.229 (0.098, 0.538)***	0.091 (0.043, 0.194)***	0.150
LA	0.778 (0.679, 0.891)***	0.822 (0.735, 0.921)***	0.337
All-cause mortality			
Total PUFA	0.845 (0.809, 0.883)***	0.828 (0.793, 0.864)***	0.616
N3FA	0.557 (0.493, 0.630)***	0.513 (0.454, 0.580)***	0.314
N6FA	0.878 (0.833, 0.926)***	0.863 (0.820, 0.909)***	0.758
N6FA/N3FA	1.025 (1.020, 1.030)***	1.016 (1.014, 1.019)***	0.002
DHA	0.229 (0.166, 0.316)***	0.167 (0.121, 0.231)***	0.097
LA	0.885 (0.841, 0.931)***	0.863 (0.821, 0.908)***	0.682

Model was adjusted for covariates including age, gender, race, educational level, income, body mass index, smoking, alcohol, IPAQ activity group, diabetes, townsend deprivation index, total serum cholesterol level, taking anti-hypertensive medication, chronic renal dysfunction or decreased eGFR. CI, confidence interval; DHA, docosahexaenoic acid; IPAQ, the International Physical Activity Questionnaire; LA, Linoleic acid; N3FA, n-3 polyunsaturated fatty acid; N6FA, n-6 polyunsaturated fatty acid; PRS, polygenic risk score; Total PUFA, N6FA + N3FA.

### Subgroup and sensitivity analysis

We conducted sex-stratified subgroup analyses (Supplementary Table S7). The results demonstrated that the protective effects of Total PUFA, N6FA, and LA on CVD events differed between men and women, with the protective associations being stronger in men (*p* < 0.001). Similarly, the protective effects of Total PUFA, N3FA, and DHA on all-cause mortality were marginally stronger in men compared to women (*p* < 0.05). In contrast, the N6FA/N3FA ratio was associated with a slightly higher risk of both CVD events (*p* < 0.05) and all-cause mortality (*p* < 0.001) in men compared to women. Sensitivity analysis employed time-dependent models to evaluate the robustness of the research results. We studied a group of people (*n* = 7,628) who received two measurements of PUFAs at the baseline and the first follow-up. After excluding individuals (*n* = 248) with prior CVD events before the second measurement (2012–2013), 7,380 participants were included in the analysis. The association between PUFAs and CVD events, CVD-related deaths, and all-cause deaths remained consistent when considering fatty acids concentrations measured at different times (Supplementary Table S8).

## Discussion

In this study, we evaluated the associations of PUFAs with CVD morbidity and mortality among hypertensive participants in the UK Biobank, as well as the moderating effects of genetic predispositions on these associations. We found that plasma PUFAs (e.g., Total PUFA, N3FA, N6FA, DHA and LA) exhibited protective associations against CVD events, CVD mortality, and all-cause mortality. However, the increased ratio of N6FA/N3FA in plasma presented an elevated risk for CVD events, CVD-related deaths, and all-cause deaths. Compared with the high CVD-PRS group, the protective associations of Total PUFA, N6FA and LA with the risk of CVD were more significant in the low CVD-PRS group, while the correlation of N6FA/N3FA with the risk of CVD and all-cause mortality was higher in individuals with low CVD-PRS.

Our observations revealed a positive association between an elevated N6FA/N3FA ratio and an increased risk of CVD. This suggests that an imbalance in the N6FA to N3FA ratio may be a relevant factor for cardiovascular health, which could inform future nutritional strategies. This result might result from the fact that N3FA can be metabolized into the so-called specific pro-resolving mediator (SPM), which may promote the resolution of inflammation; while N6FA can be converted into metabolites with pro-inflammatory properties (such as prostaglandins and leukotrienes) (25, 26). When excessive amount of N6FA was consumed, it may increase the production of pro-inflammatory metabolites, thereby intensifying the inflammatory response and mitigating the pro-resolution effects (27). This is consistent with the observed downstream N6FA metabolite harmful trend by Shi et al., where LA itself is beneficial, but its downstream metabolites such dihomo-gamma-linolenic acid (DGLA) and the DGLA/LA ratio are associated with higher risk (28). Previous studies have demonstrated that in the secondary prevention of CVD, a ratio of 4/1 between N6FA and N3FA is correlated with a 70% reduction in total mortality in the CVD population (15). However, there is currently no definite evidence to indicate what the best ratio of N6FA/N3FA is for the protection against CVD. Our study helps to address this gap by revealing a significant non-linear relationship, wherein the risks of CVD events, CVD-related mortality, and all-cause mortality were observed to be lowest at a ratio of approximately 8.70 and rose markedly with higher ratios.

Notably, DHA demonstrated particularly remarkable benefits, suggesting that DHA might possess greater advantages in preventing CVD events. Although these findings were consistent with results from other studies (29). For example, Shi et al. found a significant association between DHA and a lower risk of coronary heart disease (CHD), HR (95%CI): 0.91 (0.84, 0.98) (28). Our previous finding suggested a null association between dietary levels of DHA and CVD mortality among a US hypertensive group (18). The inconsistencies may derive from differences in the assessment of PUFAs levels and participants’ age between the two studies. Overall, our paper indicated that elevated plasma PUFAs concentrations are beneficial against CVD morbidity and mortality.

When considering genetic susceptibility of PUFAs, we did not observe any modifying effects of the PUFA – PRS on the associations between PUFAs and CVD outcomes. These findings suggest that the process of absorption, metabolism, and utilization of the fatty acids might be independent with the associations between PUFAs and CVD morbidity and mortality. Additional analysis was needed to ascertain the observed findings. However, when considering CVD – PRS, significant disparities emerged in the correlations between Total PUFA, N6FA, LA and CVD events across different genetic risk groups. Overall, blood plasma PUFAs demonstrated a more pronounced protective effects against CVD in the low CVD – PRS group compared to their high-risk counterparts, suggesting that a higher genetic risk associated with CVD may lower the protective effect of PUFA on CVD. The underlying mechanisms include the regulation of gene expression related to PUFAs metabolism, inflammatory responses, lipoprotein metabolism, and endogenous fatty acid synthesis (22, 30). Genetic variants may modulate lipid metabolism or inflammatory pathways, thereby affecting PUFA utilization. As demonstrated by Alessandro Medoro et al., *ELOVL2* SNPs directly controls the metabolic flux and tissue-enrichment efficiency of dietary PUFAs (especially EPA) into high-activity end products (DHA) by regulating activity of key elongase enzymes, and through these metabolic changes, genetic variants affect the fatty acid composition (Omega-3 index) of the cell membrane and the N6FA/N3FA balance, thereby reshaping inflammation and resolution mediators (31). This finding reinforces evidence from prior research regarding the interaction between individual genetic predisposition and PUFAs and emphasizes the necessity for developing personalized nutrition recommendations (32, 33). Interestingly, in the group with a low CVD-related PRS score, the risk ratio of N6FA/N3FA for CVD events and all-cause deaths was higher than that in the group with a high CVD-related PRS score. This might indicate that even if an individual has a low CVD-related PRS score, namely a lower genetic risk for CVD, unhealthy dietary habits, particularly a high N6FA/N3FA ratio, could still enhance the risk of CVD.

When we performed a stratified analysis, with participants categorized by gender, we observed that the protective effects of plasma Total PUFA, N6FA, and LA on CVD events varied between men and women, with more pronounced protective benefits observed in men. This might be because the study population were all above 40 years old. Male testosterone can restore vascular reactivity and endothelial function, and may reduce male cardiovascular disease (34). For women, low estrogen levels during menopause are associated with an increased risk of CVD (35). Similarly, the protective effects of plasma Total PUFA, N3FA, and DHA on all-cause mortality varied by gender, with a stronger protective effect observed in men than in women. However, for CVD-related mortality, the protective effects of these PUFAs showed no statistically significant gender differences. This phenomenon might occur because all-cause mortality is a complex and multifactorial end event involving various genetic, behavioral, and environmental factors (36). Furthermore, we have also observed that there exists a disparity in the risks of CVD events and all-cause mortality associated with plasma N6FA/N3FA levels between men and women, with a higher risk in men than in women. Regarding this specific outcome, based on previous studies where N6FA intake is higher in people’s daily lives (37), leading to an imbalance of N6FA/N3FA and an increased risk of CVD events and all-cause deaths, it is rational that the risk is increased in both sexes. However, for the slightly higher risk in men compared to women, we speculate that it might be the combined effect of testosterone in men and N6FA, thereby exacerbating the occurrence of atherosclerosis or thrombosis (38, 39).

This study report that elevated levels of plasma PUFAs, including Total PUFA, N3FA, N6FA, N6FA/N3FA, DHA, and LA in hypertensive adults within the UK Biobank are associated with decreased incidences of CVD events, CVD-related deaths, and all-cause deaths. A higher N6FA/N3FA ratio was associated with a higher risk of death, in line with the main conclusion of Zhang et al., whose was based on the general population, while our study focused precisely on patients with hypertension (16). Additionally, it is the first time that CVD – PRS and PUFA – PRS have been employed as moderators between PUFAs and CVD outcomes. This research possesses several key strengths. It employed a prospective cohort with a very large sample size and utilizes high-quality data from the UK Biobank survey. Various confounding factors, such as demographic factors, socio-economic status, medication usage, health conditions, and lifestyle choices, have been controlled to mitigate potential confounding biases. Furthermore, the measurement data of plasma PUFAs in this study are more reliable than traditional self-reported dietary records as it minimizes recall bias and measurement error to the greatest extent.

However, this study has several limitations. The hypertensive individuals in the study were predominantly white, which might constrain the generalizability of our findings to other populations. Although we have adjusted for several confounding factors, there might still exist confounding factors unaccounted for that could influence our research outcomes. Owing to the substantial proportion of missing data in the energy intake variable, no adjustment was made for daily energy intake levels in the present analysis. When using the time-dependent model, we noted that most covariates were only measured at baseline, which limited our ability to account for time-varying factors. Because plasma levels of PUFAs were only measure at the baseline for majority of the participants, we were not able to capture the variations of plasma PUFAs during the follow-up. Subsequent studies need to further investigate the complex relationship among PUFAs, genetic risk factors, and CVD, particularly N6FA/N3FA.

## Conclusion

In summary, our study of hypertensive adults from the large-scale UK Biobank cohort suggests that when evaluating the potential benefits of PUFAs on CVD, both the N6FA/N3FA ratio and genetic predisposition to CVD should be considered. Overall, higher plasma levels of Total PUFA, N3FA, N6FA, DHA, and LA were associated with reduced risks of CVD events, CVD-related mortality, and all-cause mortality. In contrast, a higher N6FA/N3FA ratio was associated with increased CVD risk. The protective associations of Total PUFA, N6FA, and LA were more pronounced in individuals with a low genetic risk of CVD compared to those with high genetic risk. These findings underscore the importance of personalized nutritional strategies for hypertensive adults, particularly regarding the balance between N6FA and N3FA, in the context of CVD prevention.

## Data Availability

Publicly available datasets were analyzed in this study. This data can be found here: UK Biobank.
