# Supplementary material for: Associations of polyunsaturated fatty acids and genetic predisposition with cardiovascular risk among hypertensive adults
Source: Front Nutr. 2025 Oct 22;12:1623962. doi: 10.3389/fnut.2025.1623962 (PMC12587298; doi:10.3389/fnut.2025.1623962)
Supplement: Supplementary file 1 [file Table_1.docx]

### Supplementary materials

| Table S1. Characteristics of hypertensive participants with PRS (CVD) data in the UK Biobank. | | |
| --- | --- | --- |
| Variables | Total (n=134,601) | |
| Age, years, Mean (SD) | 58.6(7.4) |  |
| Sex, n (%) |  |  |
| Men | 68,317(50.7) |  |
| Women | 66,284(49.3) |  |
| Race/ethnicity, n (%) |  |  |
| White | 128,086(95.1) |  |
| Other | 6,515(4.9) |  |
| Education level, n (%) |  |  |
| High | 45,503(33.8) |  |
| Moderate | 50,325(37.3) |  |
| Low | 10,219(7.5) |  |
| Annual household income, n (%) |  |  |
| <£31,000 | 70,496(52.3) |  |
| £31,000 ~ £51,999 | 22,379(16.6) |  |
| ≥£52,000 | 41,726(31.1) |  |
| Smoking status, n (%) |  |  |
| Previous | 49,868(37.0) |  |
| Current | 72,034(53.6) |  |
| Never | 12,699(9.4) |  |
| Alcohol status, n (%) |  |  |
| Previous | 5,882(4.3) |  |
| Current | 124,186(90.4) |  |
| Never | 4,533(5.3) |  |
| IPAQ activity group, n (%) |  |  |
| High | 54,829(40.8) |  |
| Moderate | 53,898(40.0) |  |
| Low | 25,874(19.2) |  |
| BMI, kg/m^2^ , Mean (SD) | 28.5(4.9) |  |
| Diabetes, n (%) |  |  |
| Yes | 9,731(7.2) |  |
| No | 124,870(92.8) |  |
| Taking anti-hypertensive medication, n (%) |  |  |
| Yes | 47,639(35.3) |  |
| No | 86,962(64.7) |  |
| Chronic renal dysfunction or decreased eGFR, n (%) |  |  |
| Yes | 24,546(18.2) |  |
| No | 110,055(81.8) |  |
| Total serum cholesterol level, mg/dL, Mean (SD) | 5.74(1.17) |  |
| Townsend deprivation index, Mean (SD) | -1.40(3.05) |  |
| Note: Categorical variables were expressed as number (n) and weighted percent (%) of the participants. Continuous variables were expressed as weighted median and SD (standard deviation). BMI: body mass index. IPAQ: the International Physical Activity Questionnaire. PRS: polygenic risk score. | | |

Table S2. Characteristics of hypertensive participants with PRS (PUFA) data in the UK Biobank.

| Variables | Total (n=26,725) | |
| --- | --- | --- |
| Age, years, Mean (SD) | 57.5(7.81) |  |
| Sex, n (%) |  |  |
| Men | 13,281(49.6) |  |
| Women | 13,444(50.3) |  |
| Race/ethnicity, n (%) |  |  |
| White | 20,278(75.8) |  |
| Other | 6,447(24.2) |  |
| Education level, n (%) |  |  |
| High | 10,392(38.8) |  |
| Moderate | 9,160(34.2) |  |
| Low | 1,930(7.2) |  |
| Annual household income, n (%) |  |  |
| <£31,000 | 13,354(50.0) |  |
| £31,000 ~ £51,999 | 4,377(16.3) |  |
| ≥£52,000 | 8,994(33.7) |  |
| Smoking status, n (%) |  |  |
| Previous | 9,313(34.8) |  |
| Current | 14,604(54.6) |  |
| Never | 2,808(10.5) |  |
| Alcohol status, n (%) |  |  |
| Previous | 2,361(8.8) |  |
| Current | 23,332(87.4) |  |
| Never | 1,032(3.8) |  |
| IPAQ activity group, n (%) |  |  |
| High | 10,740(40.2) |  |
| Moderate | 10,597(39.6) |  |
| Low | 5,388(20.2) |  |
| BMI, kg/m^2^ , Mean (SD) | 28.6(5.0) |  |
| Diabetes, n (%) |  |  |
| Yes | 2,532(9.4) |  |
| No | 24,193(90.6) |  |
| Taking anti-hypertensive medication, n (%) |  |  |
| Yes | 9,859(36.8) |  |
| No | 1,6866(63.2) |  |
| Chronic renal dysfunction or decreased eGFR, n (%) |  |  |
| Yes | 4,865(18.2) |  |
| No | 21,860(81.8) |  |
| Total serum cholesterol level, mg/dL, Mean (SD) | 5.7(1.2) |  |
| Townsend deprivation index, Mean (SD) | -0.5(3.4) |  |

Note: Categorical variables were expressed as number (n) and weighted percent (%) of the participants. Continuous variables were expressed as weighted median and SD (standard deviation). BMI: body mass index. IPAQ: the International Physical Activity Questionnaire. PRS: polygenic risk score; PRS (PUFA)：PRS (Total PUFA/N3FA/N6FA/DHA).

Table S3. Descriptive statistics of plasma fatty acid levels in PRS (CVD) hypertensive adults with PRS (CVD) data.

| Variables | Mean (SD)  n=134,601 |
| --- | --- |
| Total PUFA, mmol/L | 5.10（0.82） |
| N3FA, mmol/L | 0.55（0.23） |
| N6FA, mmol/L | 4.55（0.70） |
| N6FA/N3FA, ratio | 9.56（4.31） |
| DHA, mmol/L | 0.24（0.09） |
| LA, mmol/L | 3.48（0.70） |

Note: DHA: docosahexaenoic acid; LA: Linoleic acid; N3FA: n-3 polyunsaturated fatty acid; N6FA: n-6 polyunsaturated fatty acid; PRS: polygenic risk score; SD: standard deviation; Total PUFA: N6FA+N3FA.

Table S4. Descriptive statistics of plasma fatty acid levels in hypertensive adults with PRS (PUFA) data.

| Variables | Mean (SD)  n=26,725 | |
| --- | --- | --- |
| Total PUFA, mmol/L | 5.10（0.84） |  |
| N3FA, mmol/L | 0.55（0.23） |  |
| N6FA, mmol/L | 4.54（0.72） |  |
| N6FA/N3FA, ratio | 9.59（4.79） |  |
| DHA, mmol/L | 0.24（0.09） |  |
| LA, mmol/L | 3.48（0.73） |  |

Note: DHA: docosahexaenoic acid; LA: Linoleic acid; N3FA: n-3 polyunsaturated fatty acid; N6FA: n-6 polyunsaturated fatty acid; PRS: polygenic risk score; PRS (PUFA)：PRS (Total PUFA/N3FA/N6FA/DHA); SD: standard deviation; Total PUFA: N6FA+N3FA.

Table S5. Associations between plasma levels of fatty acids and CVD events, CVD mortality, and all-cause mortality among hypertensive adults in the UK Biobank.

| Variables | Hazard Ratio (95% CI) | | |
| --- | --- | --- | --- |
|  | CVD events | CVD mortality | All-cause mortality |
| **Total PUFA** |  |  |  |
| Continuous | 0.902(0.880,0.923)*** | 0.792(0.735,0.853)*** | 0.838(0.813,0.864)*** |
| Q1 | 1 | 1 | 1 |
| Q2 | 0.921(0.887,0.956) *** | 0.891(0.797,0.996) * | 0.857(0.818,0.899) *** |
| Q3 | 0.906(0.869,0.946) *** | 0.767(0.673,0.874) *** | 0.794(0.753,0.838) *** |
| Q4 | 0.839(0.797,0.883) *** | 0.665(0.567,0.780) *** | 0.730(0.684,0.779) *** |
| *P* for trend | ＜0.001 | ＜0.001 | ＜0.001 |
| **N3FA** |  |  |  |
| Continuous | 0.745(0.698,0.796)*** | 0.499(0.404,0.616)*** | 0.538(0.494,0.586)*** |
| Q1 | 1 | 1 | 1 |
| Q2 | 0.934(0.900,0.969) *** | 0.819(0.734,0.914) *** | 0.806(0.770,0.844) *** |
| Q3 | 0.881(0.848,0.915) *** | 0.761(0.679,0.853) *** | 0.748(0.713,0.784) *** |
| Q4 | 0.846(0.812,0.881) *** | 0.682(0.603,0.773) *** | 0.702(0.667,0.738) *** |
| P for trend | ＜0.001 | ＜0.001 | ＜0.001 |
| **N6FA** |  |  |  |
| Continuous | 0.912(0.886,0.938)*** | 0.814(0.744,0.889)*** | 0.873(0.841,0.905)*** |
| Q1 | 1 | 1 | 1 |
| Q2 | 0.945(0.910,0.982) ** | 0.848(0.757,0.951) ** | 0.891(0.849,0.934) *** |
| Q3 | 0.924(0.885,0.965) *** | 0.806(0.707,0.920) ** | 0.836(0.792,0.883) *** |
| Q4 | 0.874(0.830,0.921) *** | 0.708(0.603,0.833) *** | 0.820(0.768,0.876) *** |
| P for trend | ＜0.001 | ＜0.001 | ＜0.001 |
| **N6FA/N3FA** |  |  |  |
| Continuous | 1.011(1.008,1.013)*** | 1.018(1.012,1.023)*** | 1.018(1.016,1.020)*** |
| Q1 | 1 | 1 | 1 |
| Q2 | 1.019(0.982,1.058) | 1.068(0.949,1.203) | 1.039(0.990,1.090) |
| Q3 | 1.085(1.045,1.126) *** | 1.177(1.047,1.324) ** | 1.122(1.069,1.177) *** |
| Q4 | 1.156(1.113,1.201) *** | 1.364(1.214,1.533) *** | 1.366(1.303,1.433) *** |
| P for trend | ＜0.001 | ＜0.001 | ＜0.001 |
| **DHA** |  |  |  |
| Continuous | 0.425(0.357,0.506)*** | 0.136(0.077,0.239)*** | 0.197(0.157,0.247)*** |
| Q1 | 1 | 1 | 1 |
| Q2 | 0.922(0.889,0.956) *** | 0.809(0.727,0.901) *** | 0.834(0.797,0.873) *** |
| Q3 | 0.882(0.850,0.916) *** | 0.730(0.651,0.820) *** | 0.777(0.741,0.815) *** |
| Q4 | 0.826(0.794,0.861) *** | 0.667(0.588,0.756) *** | 0.709(0.674,0.747) *** |
| P for trend | ＜0.001 | ＜0.001 | ＜0.001 |
| **LA** |  |  |  |
| Continuous | 0.914(0.889,0.939)*** | 0.807(0.741,0.880)*** | 0.877(0.846,0.908)*** |
| Q1 | 1 | 1 | 1 |
| Q2 | 0.940(0.905,0.976) ** | 0.874(0.780,0.979) * | 0.895(0.854,0.939) *** |
| Q3 | 0.934(0.895,0.975) ** | 0.760(0.666,0.868) *** | 0.829(0.785,0.875) *** |
| Q4 | 0.877(0.833,0.923) *** | 0.713(0.609,0.835) *** | 0.808(0.758,0.862) *** |
| P for trend | ＜0.001 | ＜0.001 | ＜0.001 |

Note: Cox proportional hazrds model was employed to assess the associations with adjustment for covariates including age, gender, race, educational level, income, body mass index, smoking, alcohol, IPAQ activity group, diabetes, townsend deprivation index, total serum cholesterol level, taking anti-hypertensive medication, chronic renal dysfunction or decreased eGFR. CI: confidence interval; DHA: docosahexaenoic acid; IPAQ: the International Physical Activity Questionnaire. LA: Linoleic acid; N3FA: n-3 polyunsaturated fatty acid; N6FA: n-6 polyunsaturated fatty acid; Total PUFA: N6FA+N3FA. *: P＜0.05；**：P＜0.01；***：P＜0.001.

Table S6. Interactions between plasma levels of PUFAs and PUFA-PRS on CVD events, CVD mortality, all-cause mortality in hypertensive adults.

|  | Hazards ratio (95%CI) | | *P_interaction_* |
| --- | --- | --- | --- |
|  | PRS-Low | PRS-High |  |
| CVD events |  |  |  |
| Total PUFA | 0.972(0.896,1.054) | 0.940(0.874,1.011) | 0.612 |
| N3FA | 0.958(0.773,1.187) | 0.705(0.571,0.870)** | 0.093 |
| N6FA | 1.014(0.923,1.115) | 0.946(0.868,1.031) | 0.364 |
| DHA | 0.607(0.349,1.056) | 0.307(0.174,0.540)*** | 0.157 |
| CVD mortality |  |  |  |
| Total PUFA | 0.778(0.601,1.007) | 0.790(0.630,0.991)* | 0.559 |
| N3FA | 0.398(0.189,0.841)* | 0.477( 0.242,0.940)* | 0.577 |
| N6FA | 0.884(0.657,1.189) | 0.781(0.598,1.020) | 0.776 |
| DHA | 0.061(0.009, 0.414)** | 0.047(0.007,0.312)** | 0.949 |
| All-cause mortality |  |  |  |
| Total PUFA | 0.824(0.742,0.915)*** | 0.873(0.794,0.960)** | 0.565 |
| N3FA | 0.551(0.412,0.736)*** | 0.649(0.494,0.852)** | 0.265 |
| N6FA | 0.849(0.750,0.961)** | 0.897(0.803,1.002) | 0.282 |
| DHA | 0.182(0.086,0.385)*** | 0.281(0.136,0.580)*** | 0.671 |

Note: Model was adjusted for covariates including age, gender, race, educational level, income, body mass index, smoking, alcohol, IPAQ activity group, diabetes, townsend deprivation index, total serum cholesterol level, taking anti-hypertensive medication, chronic renal dysfunction or decreased eGFR. CI: confidence interval; DHA: docosahexaenoic acid; IPAQ: the International Physical Activity Questionnaire.; N3FA: n-3 polyunsaturated fatty acid; N6FA: n-6 polyunsaturated fatty acid.; PRS: polygenic risk score; Total PUFA: N6FA+N3FA. *: P＜0.05；**：P＜0.01；***：P＜0.001.

Table S7. Associations between plasma levels of PUFAs and CVD events, CVD mortality, all-cause mortality among male and female hypertensive adults.

|  | Hazards ratio (95%CI) | | *P*-value |
| --- | --- | --- | --- |
|  | Men | Women |  |
| CVD events |  |  |  |
| Total PUFA | 0.8929(0.8662,0.9203)*** | 0.9148(0.8798,0.9511)*** | <0.001 |
| N3FA | 0.7236(0.6643,0.7881)*** | 0.7775(0.7012,0.8622)*** | 0.763 |
| N6FA | 0.9017(0.8695,0.9351)*** | 0.9261(0.8836,0.9706)** | <0.001 |
| N6FA/N3FA | 1.0127(1.0096,1.0158)*** | 1.0072(1.0034,1.0111)*** | 0.022 |
| DHA | 0.4318(0.3458,0.5391)*** | 0.4416(0.3337,0.5843)*** | 0.1403 |
| LA | 0.9011(0.8699,0.9335)*** | 0.9360(0.8946,0.9793)** | <0.001 |
| CVD mortality |  |  |  |
| Total PUFA | 0.7884(0.7222,0.8607)*** | 0.8059(0.7026,0.9244)** | 0.270 |
| N3FA | 0.4519(0.3488,0.5856)*** | 0.6184(0.4278,0.8940)* | 0.262 |
| N6FA | 0.8178(0.7361,0.9085)*** | 0.8068(0.6840,0.9518)* | 0.121 |
| N6FA/N3FA | 1.0223(1.0158,1.0289)*** | 1.0078(0.9938,1.0219) | 0.060 |
| DHA | 0.1434(0.0731,0.2814)*** | 0.1320(0.0475,0.3667)*** | 0.622 |
| LA | 0.7968(0.7191,0.8829)*** | 0.8389(0.7154,0.9837)* | 0.265 |
| All-cause mortality |  |  |  |
| Total PUFA | 0.7952(0.7643,0.8273)*** | 0.9081(0.8656,0.9526)*** | 0.020 |
| N3FA | 0.4482(0.3991,0.5034)*** | 0.6844(0.6021,0.7780)*** | <0.001 |
| N6FA | 0.8306(0.7921,0.8710)*** | 0.9409(0.8883,0.9965)* | 0.127 |
| N6FA/N3FA | 1.0232(1.0204,1.0261)*** | 1.0131(1.0096,1.0167)*** | <0.001 |
| DHA | 0.1443(0.1068,0.1950)*** | 0.3101(0.2190,0.4389)*** | 0.003 |
| LA | 0.8338(0.7962,0.8731)*** | 0.9440(0.8932,0.9977)* | 0.085 |

Note: Model was adjusted for covariates including age, gender, race, educational level, income, body mass index, smoking, alcohol, IPAQ activity group, diabetes, townsend deprivation index, total serum cholesterol level, taking anti-hypertensive medication, chronic renal dysfunction or decreased eGFR. CI: confidence interval; DHA: docosahexaenoic acid; IPAQ: the International Physical Activity Questionnaire; LA: Linoleic acid; N3FA: n-3 polyunsaturated fatty acid; N6FA: n-6 polyunsaturated fatty acid; Total PUFA: N6FA+N3FA. *: P＜0.05；**：P＜0.01；***：P＜0.001.

Table S8. Associations of PUFAs with CVD events, CVD mortality, and all-cause mortality among hypertensive adults analyzed by time-dependent cox proportional hazards model.

| Variables | Hazards ratio (95%CI) | | | |  |
| --- | --- | --- | --- | --- | --- |
|  | CVD events | CVD mortality | All-cause mortality | |  |
| Total PUFA | 0.8450(0.7880,0.9061)*** | 0.7211(0.5712,0.9102)** | | 0.7316(0.6616,0.8089)*** | |
| N3FA | 0.6770(0.5497,0.8337)*** | 0.4659(0.2267,0.9574)* | | 0.4559(0.3340,0.6224)*** |  |
| N6FA | 0.8395(0.7725,0.9123)*** | 0.7087(0.5368,0.9356)* | | 0.7281(0.6462,0.8204)*** |  |
| N6FA/N3FA | 1.0185(1.0084,1.0288)*** | 1.0280(0.9948,1.0620) | | 1.0312(1.0178,1.0448)*** |  |
| DHA | 0.3369(0.1941,0.5848)*** | 0.1028(0.0148,0.7129)* | | 0.1637(0.0726,0.3694)*** |  |
| LA | 0.8510(0.7852,0.9223)*** | 0.6591(0.5030,0.8636)** | | 0.7479(0.6663,0.8395)*** |  |

Note: Time-dependent cox proportional hazrds model was employed to assess the associations with adjustment for covariates including age, gender, race, educational level, income, body mass index, smoking, alcohol, IPAQ activity group, diabetes, Townsend Deprivation Index, total serum cholesterol level, taking anti-hypertensive medication, chronic renal dysfunction or decreased eGFR. CI: confidence interval; DHA: docosahexaenoic acid; IPAQ: the International Physical Activity Questionnaire.; LA: Linoleic acid; N3FA: n-3 polyunsaturated fatty acid; N6FA: n-6 polyunsaturated fatty acid. Total PUFA: N6FA+N3FA. *: P＜0.05；**：P＜0.01；***：P＜0.001.

| Table S9. Associations between plasma levels of fatty acids and myocardial infarction events among hypertensive adults in the UK Biobank. | |
| --- | --- |
| Variables | Hazard Ratio (95% CI) |
| Total PUFA | 0.946(0.906,0.987)** |
| N3FA | 0.767(0.682,0.863)*** |
| N6FA | 0.971(0.923,1.021) |
| N6FA/N3FA | 1.010(1.006,1.015)*** |
| DHA | 0.274(0.199,0.377)*** |
| LA | 0.995(0.947,1.045) |
| Note: Cox proportional hazrds model was employed to assess the associations with adjustment for covariates including age, gender, race, educational level, income, body mass index, smoking, alcohol, IPAQ activity group, diabetes, townsend deprivation index, total serum cholesterol level, taking anti-hypertensive medication, chronic renal dysfunction or decreased eGFR. CI: confidence interval; DHA: docosahexaenoic acid; IPAQ: the International Physical Activity Questionnaire. LA: Linoleic acid; N3FA: n-3 polyunsaturated fatty acid; N6FA: n-6 polyunsaturated fatty acid; Total PUFA: N6FA+N3FA. *: P＜0.05；**：P＜0.01；***：P＜0.001. | |

| Table S10. Associations between plasma levels of fatty acids and heart failure events among hypertensive adults in the UK Biobank. | |
| --- | --- |
| Variables | Hazard Ratio (95% CI) |
| Total PUFA | 0.882(0.842,0.923)*** |
| N3FA | 0.587(0.516,0.667)*** |
| N6FA | 0.924(0.874,0.976)** |
| N6FA/N3FA | 1.014(1.011,1.018)*** |
| DHA | 0.235(0.167,0.330)*** |
| LA | 0.911(0.863,0.961)*** |
| Note: Cox proportional hazrds model was employed to assess the associations with adjustment for covariates including age, gender, race, educational level, income, body mass index, smoking, alcohol, IPAQ activity group, diabetes, townsend deprivation index, total serum cholesterol level, taking anti-hypertensive medication, chronic renal dysfunction or decreased eGFR. CI: confidence interval; DHA: docosahexaenoic acid; IPAQ: the International Physical Activity Questionnaire. LA: Linoleic acid; N3FA: n-3 polyunsaturated fatty acid; N6FA: n-6 polyunsaturated fatty acid; Total PUFA: N6FA+N3FA. *: P＜0.05；**：P＜0.01；***：P＜0.001. | |

| Table S11. Associations between plasma levels of fatty acids and atrial fibrillation and flutter events among hypertensive adults in the UK Biobank. | |
| --- | --- |
| Variables | Hazard Ratio (95% CI) |
| Total PUFA | 0.884(0.856,0.914)*** |
| N3FA | 0.782(0.716,0.854)*** |
| N6FA | 0.878(0.844,0.913)*** |
| N6FA/N3FA | 1.008(1.004,1.011)*** |
| DHA | 0.722(0.574,0.908)** |
| LA | 0.869(0.836,0.902)*** |
| Note: Cox proportional hazrds model was employed to assess the associations with adjustment for covariates including age, gender, race, educational level, income, body mass index, smoking, alcohol, IPAQ activity group, diabetes, townsend deprivation index, total serum cholesterol level, taking anti-hypertensive medication, chronic renal dysfunction or decreased eGFR. CI: confidence interval; DHA: docosahexaenoic acid; IPAQ: the International Physical Activity Questionnaire. LA: Linoleic acid; N3FA: n-3 polyunsaturated fatty acid; N6FA: n-6 polyunsaturated fatty acid; Total PUFA: N6FA+N3FA. *: P＜0.05；**：P＜0.01；***：P＜0.001. | |
| Table S12. Associations between plasma levels of fatty acids and stroke events among hypertensive adults in the UK Biobank. | |
| Variables | Hazard Ratio (95% CI) |
| Total PUFA | 0.835(0.752,0.927)*** |
| N3FA | 0.676(0.508,0.899)** |
| N6FA | 0.832(0.734,0.943)** |
| N6FA/N3FA | 1.015(1.007,1.024)*** |
| DHA | 0.288(0.135,0.617)** |
| LA | 0.863(0.765,0.973)* |
| Note: Cox proportional hazrds model was employed to assess the associations with adjustment for covariates including age, gender, race, educational level, income, body mass index, smoking, alcohol, IPAQ activity group, diabetes, townsend deprivation index, total serum cholesterol level, taking anti-hypertensive medication, chronic renal dysfunction or decreased eGFR. CI: confidence interval; DHA: docosahexaenoic acid; IPAQ: the International Physical Activity Questionnaire. LA: Linoleic acid; N3FA: n-3 polyunsaturated fatty acid; N6FA: n-6 polyunsaturated fatty acid; Total PUFA: N6FA+N3FA. *: P＜0.05；**：P＜0.01；***：P＜0.001. | |

| Table S13. Associations between plasma levels of fatty acids and atherosclerosis events among hypertensive adults in the UK Biobank. | |
| --- | --- |
| Variables | Hazard Ratio (95% CI) |
| Total PUFA | 0.827(0.750,0.911)*** |
| N3FA | 0.668(0.510,0.876)** |
| N6FA | 0.820(0.729,0.923)*** |
| N6FA/N3FA | 1.014(1.006,1.023)*** |
| DHA | 0.175(0.083,0.365)*** |
| LA | 0.799(0.712,0.896)*** |
| Note: Cox proportional hazrds model was employed to assess the associations with adjustment for covariates including age, gender, race, educational level, income, body mass index, smoking, alcohol, IPAQ activity group, diabetes, townsend deprivation index, total serum cholesterol level, taking anti-hypertensive medication, chronic renal dysfunction or decreased eGFR. CI: confidence interval; DHA: docosahexaenoic acid; IPAQ: the International Physical Activity Questionnaire. LA: Linoleic acid; N3FA: n-3 polyunsaturated fatty acid; N6FA: n-6 polyunsaturated fatty acid; Total PUFA: N6FA+N3FA. *: P＜0.05；**：P＜0.01；***：P＜0.001. | |
| Table S14. Associations between plasma levels of fatty acids and aneurysm events among hypertensive adults in the UK Biobank. | |
| Variables | Hazard Ratio (95% CI) |
| Total PUFA | 0.8166(0.7462,0.8935)*** |
| N3FA | 0.7103(0.5548,0.9094)** |
| N6FA | 0.7970(0.7151,0.8883)*** |
| N6FA/N3FA | 1.0094(0.9996,1.0192) |
| DHA | 0.2269(0.1168,0.4409)*** |
| LA | 0.8318(0.7490,0.9237)*** |
| Note: Cox proportional hazrds model was employed to assess the associations with adjustment for covariates including age, gender, race, educational level, income, body mass index, smoking, alcohol, IPAQ activity group, diabetes, townsend deprivation index, total serum cholesterol level, taking anti-hypertensive medication, chronic renal dysfunction or decreased eGFR. CI: confidence interval; DHA: docosahexaenoic acid; IPAQ: the International Physical Activity Questionnaire. LA: Linoleic acid; N3FA: n-3 polyunsaturated fatty acid; N6FA: n-6 polyunsaturated fatty acid; Total PUFA: N6FA+N3FA. *: P＜0.05；**：P＜0.01；***：P＜0.001. | |

| Table S15. Associations between plasma levels of fatty acids and arteriovenous thrombosis or embolism events among hypertensive adults in the UK Biobank. | |
| --- | --- |
| Variables | Hazard Ratio (95% CI) |
| Total PUFA | 0.8566(0.7609,0.9644)* |
| N3FA | 0.6628(0.4763,0.9224)* |
| N6FA | 0.8651(0.7503,0.9974)* |
| N6FA/N3FA | 1.0155(1.0066,1.0244)*** |
| DHA | 0.2247(0.0923,0.5474)** |
| LA | 0.8486(0.7390,0.9744)* |
| Note: Cox proportional hazrds model was employed to assess the associations with adjustment for covariates including age, gender, race, educational level, income, body mass index, smoking, alcohol, IPAQ activity group, diabetes, townsend deprivation index, total serum cholesterol level, taking anti-hypertensive medication, chronic renal dysfunction or decreased eGFR. CI: confidence interval; DHA: docosahexaenoic acid; IPAQ: the International Physical Activity Questionnaire. LA: Linoleic acid; N3FA: n-3 polyunsaturated fatty acid; N6FA: n-6 polyunsaturated fatty acid; Total PUFA: N6FA+N3FA. *: P＜0.05；**：P＜0.01；***：P＜0.001. | |
| Table S16. Associations between plasma levels of fatty acids and hypertensive heart disease events among hypertensive adults in the UK Biobank. | |
| Variables | Hazard Ratio (95% CI) |
| Total PUFA | 0.9848(0.8248,1.1759) |
| N3FA | 0.7375(0.4463,1.2189) |
| N6FA | 1.0354(0.8370,1.2807) |
| N6FA/N3FA | 1.0153(1.0029,1.028)* |
| DHA | 0.3767(0.0985,1.4402) |
| LA | 0.9835(0.7991,1.2104) |
| Note: Cox proportional hazrds model was employed to assess the associations with adjustment for covariates including age, gender, race, educational level, income, body mass index, smoking, alcohol, IPAQ activity group, diabetes, townsend deprivation index, total serum cholesterol level, taking anti-hypertensive medication, chronic renal dysfunction or decreased eGFR. CI: confidence interval; DHA: docosahexaenoic acid; IPAQ: the International Physical Activity Questionnaire. LA: Linoleic acid; N3FA: n-3 polyunsaturated fatty acid; N6FA: n-6 polyunsaturated fatty acid; Total PUFA: N6FA+N3FA. *: P＜0.05；**：P＜0.01；***：P＜0.001. | |


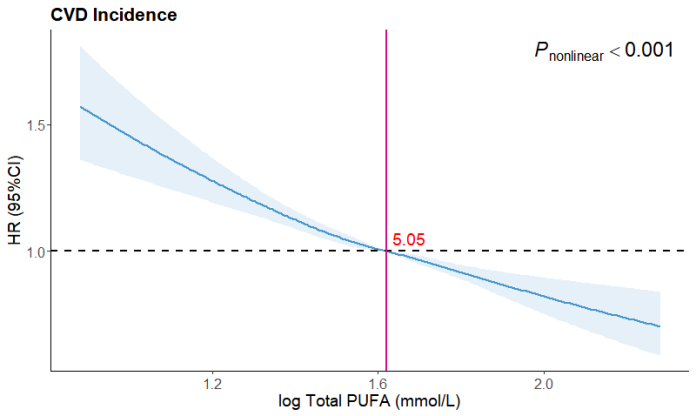

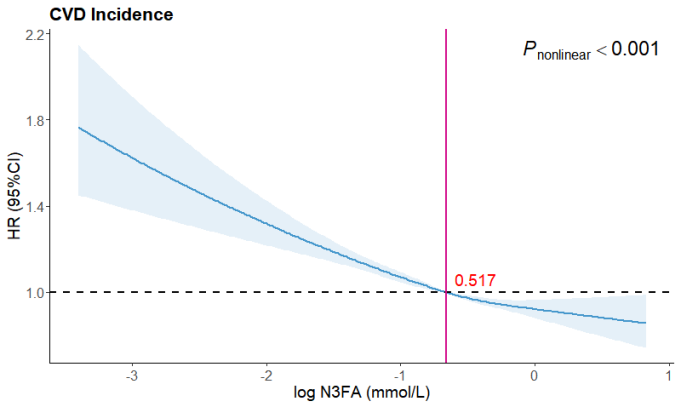

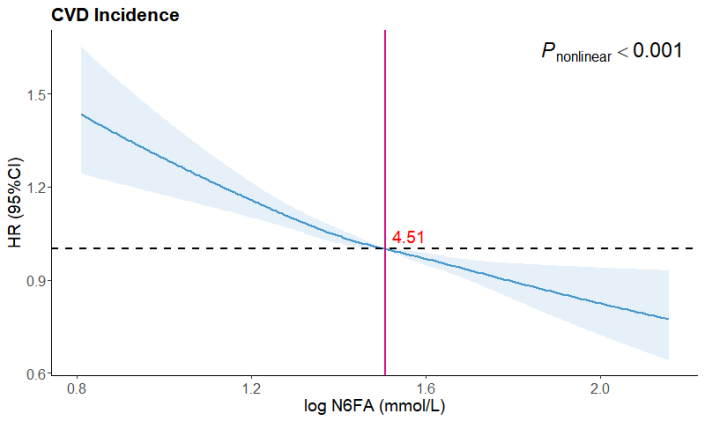


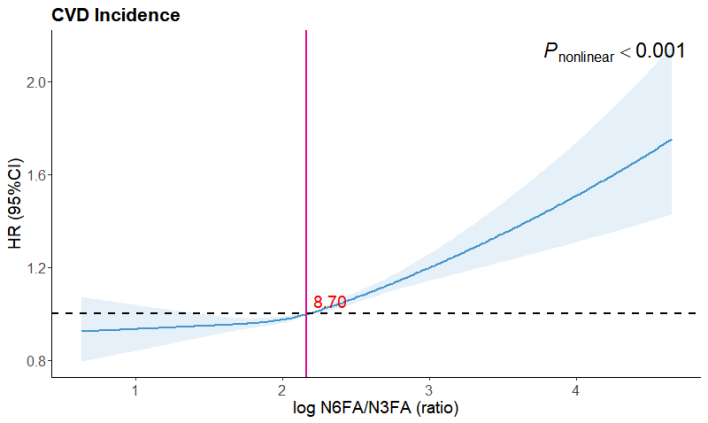

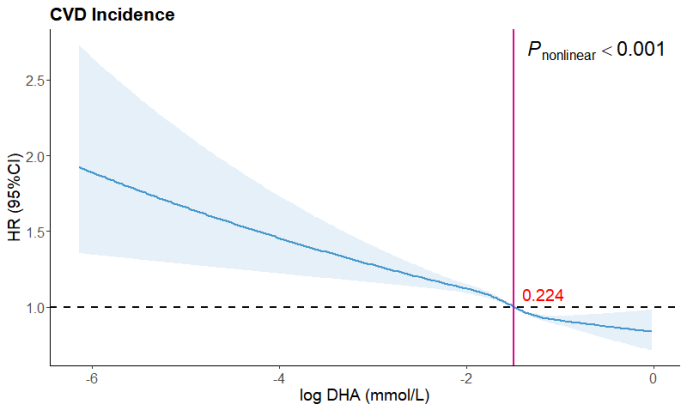

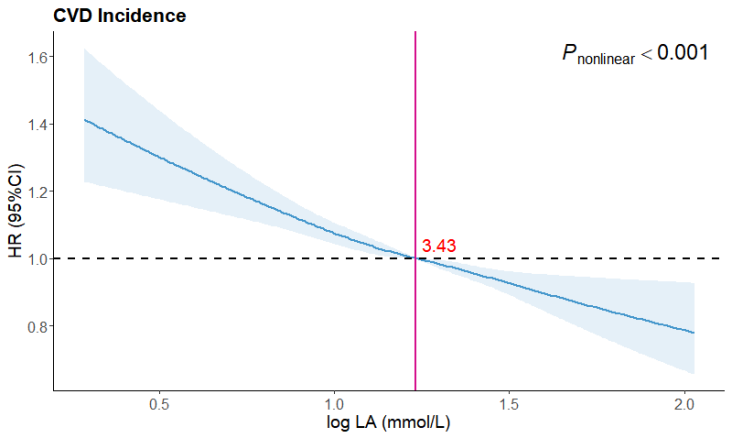


Figure S1. Non-linear associations between blood levels of fatty acids and CVD events among hypertensive adults in the UK Biobank.

Note: Cox proportional hazrds model was employed to assess the associations with adjustment for age, gender, race, educational level, income, body mass index, smoking, alcohol, IPAQ activity group, diabetes, townsend deprivation index, total serum cholesterol level, takinganti-hypertensive medication, chronic renal dysfunction or decreased eGFR. The black dashed line represents HR=1. The pink solid line represents the level of fatty acids when HR=1.


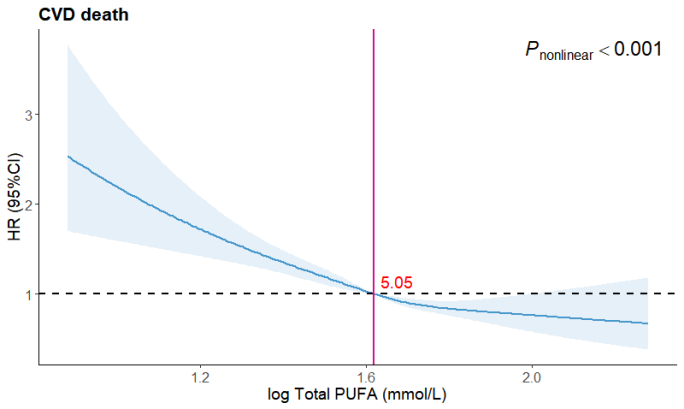

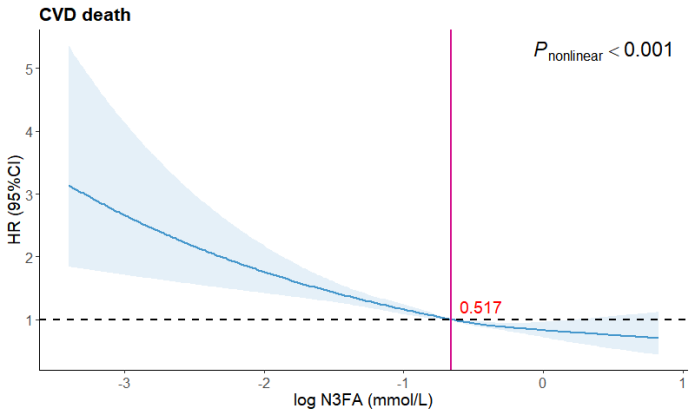

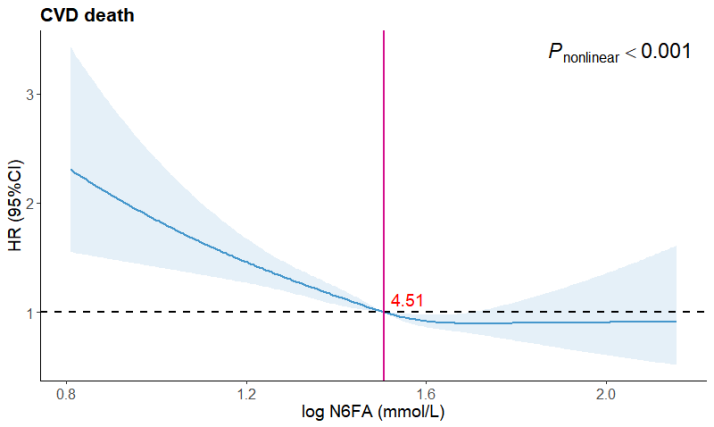

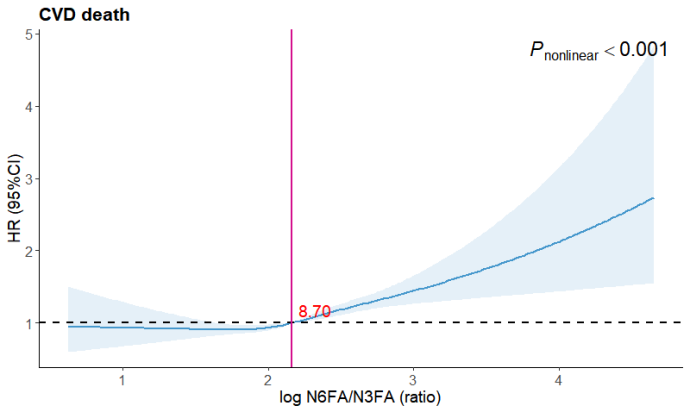

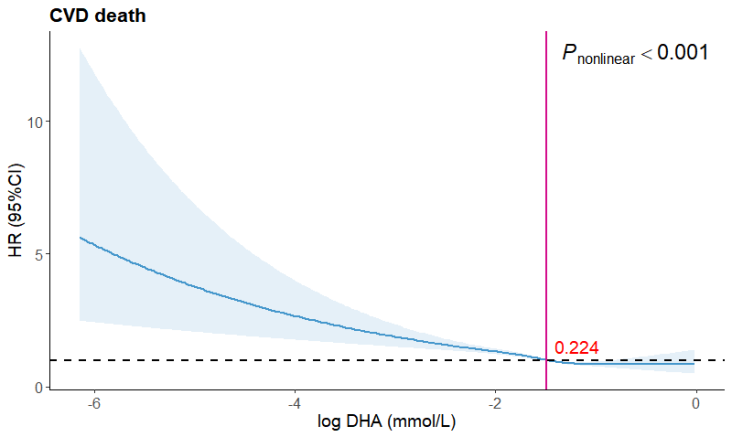

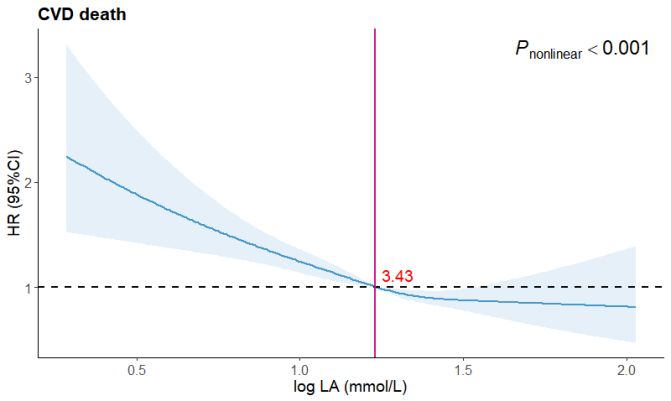


Figure S2. Non-linear associations between blood levels of fatty acids and CVD mortality among hypertensive adults in the UK Biobank.

Note: Cox proportional hazrds model was employed to assess the associations with adjustment for age, gender, race, educational level, income, body mass index, smoking, alcohol, IPAQ activity group, diabetes, townsend deprivation index, total serum cholesterol level, takinganti-hypertensive medication, chronic renal dysfunction or decreased eGFR. The black dashed line represents HR=1. The pink solid line represents the level of fatty acids when HR=1.
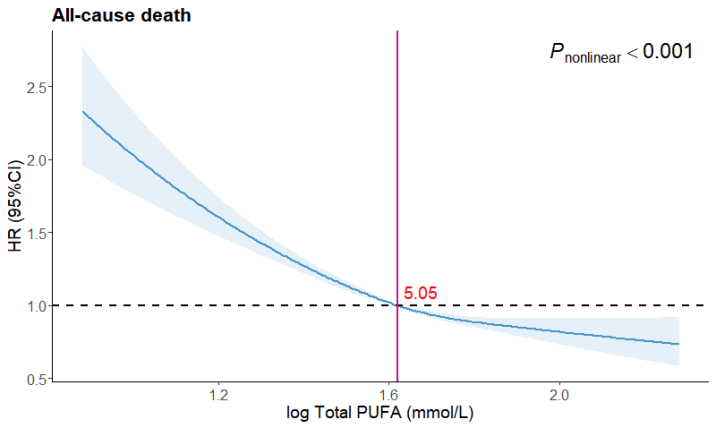

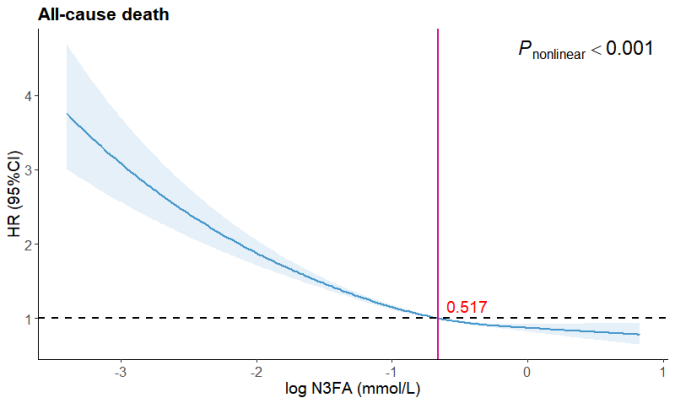

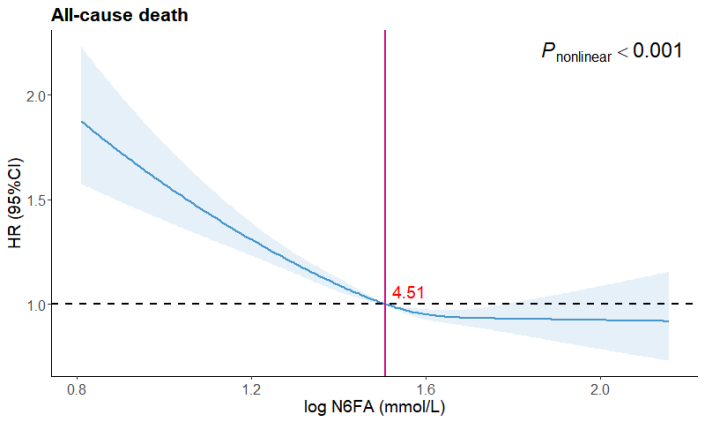


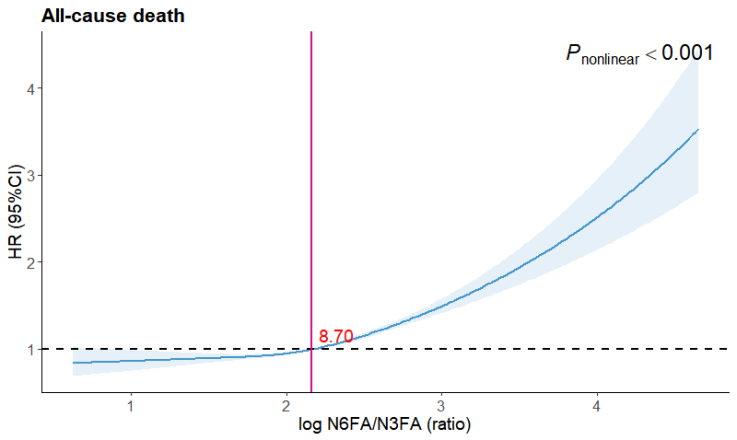

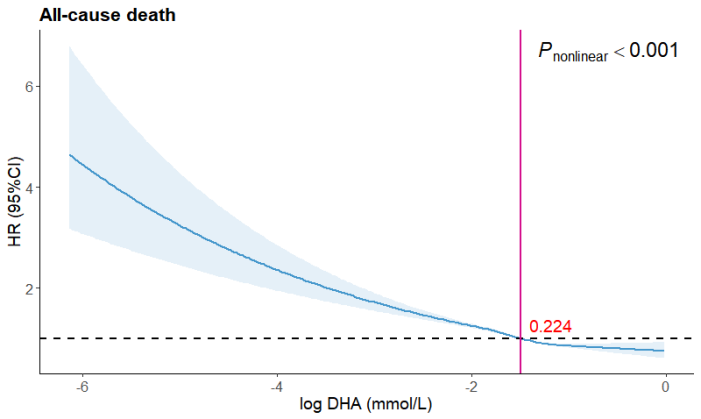

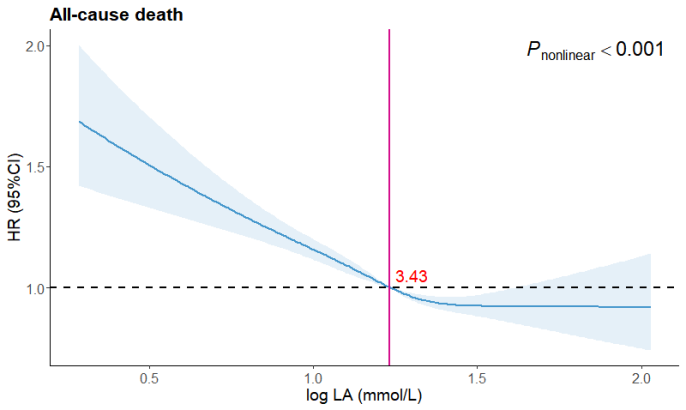


Figure S3. Non-linear associations between blood levels of fatty acids and all-cause mortality among hypertensive adults in the UK Biobank.

Note: Cox proportional hazrds model was employed to assess the associations with adjustment for age, gender, race, educational level, income, body mass index, smoking, alcohol, IPAQ activity group, diabetes, townsend deprivation index, total serum cholesterol level, takinganti-hypertensive medication, chronic renal dysfunction or decreased eGFR. The black dashed line represents HR=1. The pink solid line represents the level of fatty acids when HR=1.
